# Nanoporous Carbon Materials Derived from Zanthoxylum Bungeanum Peel and Seed for Electrochemical Supercapacitors

**DOI:** 10.3390/nano14100836

**Published:** 2024-05-09

**Authors:** Peng Jia, Ziming Wang, Xinru Wang, Ke Qin, Jiajing Gao, Jiazhen Sun, Guangmei Xia, Tao Dong, Yanyan Gong, Zhenjiang Yu, Jinyang Zhang, Honglei Chen, Shengdan Wang

**Affiliations:** 1 Key Laboratory of Pulp and Paper Science & Technology of Ministry of Education/Shandong Province, State Key Laboratory of Biobased Material and Green Papermaking, Faculty of Light Industry, Qilu University of Technology (Shandong Academy of Sciences), Jinan 250353, China; skl_jiapeng@qlu.edu.cn (P.J.); 202291040062@stu.qlu.edu.cn (Z.W.); 202291040040@stu.qlu.edu.cn (X.W.); 202182060027@stu.qlu.edu.cn (K.Q.); 202182040004@stu.qlu.edu.cn (J.G.); jiazhensun@qlu.edu.cn (J.S.); gmxia@qlu.edu.cn (G.X.); dongt_skl@qlu.edu.cn (T.D.); 531995@qlu.edu.cn (Y.G.); yuzhen-jiang@hit.edu.cn (Z.Y.); wsd6849@qlu.edu.cn (S.W.); 2 Key Laboratory of Advanced Energy Materials Chemistry (Ministry of Education), Renewable Energy Conversion and Storage Center (RECAST), College of Chemistry, Nankai University, Tianjin 300071, China

**Keywords:** biomass, zanthoxylum bungeanum, nanoporous carbon, supercapacitor, pyrolysis, activation

## Abstract

In order to prepare biomass-derived carbon materials with high specific capacitance at a low activation temperature (≤700 °C), nanoporous carbon materials were prepared from zanthoxylum bungeanum peels and seeds via the pyrolysis and KOH-activation processes. The results show that the optimal activation temperatures are 700 °C and 600 °C for peels and seeds. Benefiting from the hierarchical pore structure (micropores, mesopores, and macropores), the abundant heteroatoms (N, S, and O) containing functional groups, and plentiful electrochemical active sites, the PAC-700 and SAC-600 derive the large capacities of ~211.0 and ~219.7 F g^−1^ at 1.0 A g^−1^ in 6 M KOH within the three-electrode configuration. Furthermore, the symmetrical supercapacitors display a high energy density of 22.9 and 22.4 Wh kg^−1^ at 7500 W kg^−1^ assembled with PAC-700 and SAC-600, along with exceptional capacitance retention of 99.1% and 93.4% over 10,000 cycles at 1.0 A g^−1^. More significantly, the contribution here will stimulate the extensive development of low-temperature activation processes and nanoporous carbon materials for electrochemical energy storage and beyond.

## 1. Introduction

In the rapidly evolving field of technology, the escalating energy demand is leading to the rapid depletion of non-renewable resources on Earth. Consequently, exploring viable clean energy sources has become a focal point for researchers [1,2,3]. Many renewable resources, such as wind, tidal, and solar energy, are dependent on daytime duration and weather conditions, which require special energy storage devices due to their inherent fluctuations [4]. Supercapacitors have garnered widespread attention for their high power density, fast charge-discharge performance, and long lifespan. However, the primary challenge associated with supercapacitors is achieving both high specific energy and substantial specific power simultaneously [5,6,7].

Regarding the energy storage mechanism of supercapacitors, which can be categorized as electrical double-layer capacitors (EDLC) via surface electrostatic ion adsorption at the electrode/electrolyte interface and pseudocapacitors by the electron transfer between the electrolyte ion and electrode surface, the electrode materials play a crucial role [8]. Therefore, the development of electrode materials becomes particularly important. The rational utilization of biological resources contributes to energy conservation and enhances the value of these resources, making them excellent precursors for supercapacitors. Due to their unique chemical compositions and structural characteristics, numerous bio-derived carbon materials, including seafood shells [9], flowers [10], mushrooms [11], straw [12], cellulose nanocrystals [13], blackberry seeds [14], et al. [15], are currently being applied in the new energy field. To detail, the N, S, P, and O heteroatoms in the carbon matrix from the bio-derived precursors can change the neighboring C-C bond polarity to improve the wettability and conductivity of the carbon surface [4,16]. Moreover, the number of pores in carbon materials can increase the transmission of electrolytes, improving their capacitances [17,18]. However, the suitable carbonization and activation process plays a vital role in preserving the structural features of the biomass precursor, rationalizing pore distribution, retaining heteroatom content, and enhancing the carbon material’s ion adsorption, and pseudocapacitive reaction is crucial.

Biomass-derived precursors often contain a significant amount of heteroatoms, avoiding the need for subsequent doping of pure carbon materials and simplifying the experimental process. Nevertheless, improper carbonization and activation processes can lead to the collapse of precursor pore structures and the loss of heteroatoms. Therefore, suitable carbonization and activation methods are crucial for obtaining outstanding electrode materials. Commonly used activating agents include potassium hydroxide (KOH) [19], sodium hydroxide (NaOH) [20], sodium carbonate (Na_2_CO_3_) [21], zinc chloride (ZnCl_2_) [22], and potassium carbonate (K_2_CO_3_) [23], among others [24]. KOH has been extensively studied due to its reaction with biomass precursors, generating soluble K salts, significant CO_2_, and water vapor, forming abundant pore structures [25,26]. Hence, controlling the amount of KOH and the carbonization process is essential to balancing carbon materials’ graphitization degree and heteroatom content.

However, biomass-derived carbon materials usually have a lower specific surface area and lower specific capacitance at a lower activation temperature (≤700 °C). Additionally, the zanthoxylum bungeanum shells and seed-derived carbon materials have not been studied. Therefore, this work focuses on the structural characterization and performance analysis of KOH-activated zanthoxylum bungeanum shells and seed-derived carbon (PAC and SAC) materials, elucidating the relationship between their structures and performances. In detail, the optimal activation temperatures are 700 °C and 600 °C for peels and seeds (PAC-700 and SAC-600). SAC-600 derives the large capacities of ~219.7 F g^−1^ larger than that of PAC-700 (~211.0 F g^−1^) at 1.0 A g^−1^ in 6 M KOH within the three-electrode configuration. These characteristics strongly represent the promise of biomass-derived nanoporous carbon materials for supercapacitors.

## 2. Materials and Methods

### 2.1. Materials

The peel and seed of Dahongpao zanthoxylum bungeanum were purchased from Shaanxi Weikang Biotechnology Co., Ltd. (Hancheng, China). The chemical composition of zanthoxylum bungeanum peels/seeds are as follows: 62.35%/55.61% C, 7.94%/9.37% N, 27.35%/31.84% O, and 2.36%/3.18% S. The potassium hydroxide (KOH), acetylene black, polyvinylidene fluoride (PVDF), and concentrated hydrochloric acid (36–38%) were purchased from Sinopharm Chemical Reagent Co., Ltd. (Shanghai, China). The spectroscopically pure potassium bromide (KBr) was purchased from Sigma-Aldrich Shanghai Trading Co., Ltd. (Shanghai, China). The deionized water (18.25 MΩ·cm) was prepared and collected by using an ultrapure water purification system (TSS1-10UV2/UF, Taiping-M, Ningbo, China). All chemical reagents were used without further purification.

### 2.2. Preparation of Nanoporous Carbon Materials

Typically, the nanoporous carbon materials were prepared via pyrolysis and activation processes. Prior to the activation process, the zanthoxylum bungeanum peel and seed were pre-processed according to the subsequently described steps. Firstly, the zanthoxylum bungeanum peel and seed were separated manually and washed with deionized water to remove clay and impurities. Secondly, the separated peel and seed were dried at 80 °C for 24 h in an oven. Thirdly, the dried peel and seed were crushed into granules using a grinder. Finally, the crushed samples were heated to 500 °C with a heating rate of 10 °C/min and pyrolyzed for 2 h in a quartz tube under an argon flow rate of 100 mL/min. It should be noted that the volume of biomass materials should be less than that of the quartz tube at 1/50 during the pyrolysis. This is because colossal amounts of gases were produced during the pyrolysis, easily leading to gas leakage, the rupture of the quartz tube, and conflagration. Moreover, an absorption device was put at the outlet, and an outlet pipe with an internal diameter of ~6 mm was used to prevent the blockage of bio-oil in the outlet pipe.

After the pyrolysis, the obtained carbon materials (namely pyrolyzed zanthoxylum bungeanum peel or seed) were activated with KOH according to the following procedures. Herein, the zanthoxylum bungeanum peel was chosen as an example for understanding. Firstly, the pyrolyzed zanthoxylum bungeanum peel of 2.0 g was added to a 10 wt.% KOH aqueous solution of 20 g in a beaker of 100 mL and the additional four beakers were also prepared for the above step. Subsequently, all beakers were put on a magnetic stirrer for 2 h of agitation. Afterward, all beakers were put into a water bath, and the mixtures were evaporated and dried at 80 °C. After adequate grinding, the pyrolyzed zanthoxylum bungeanum peel was activated at the target temperatures (X = 600, 700, 800, 900, and 1000 °C) for 2 h under the same condition of pyrolysis, respectively. After that, the obtained samples were washed with 2.0 mol·L^−1^ HCl and then washed with deionized water several times until the pH of the filtrate was neutral. Whereafter, the activated carbon materials were dried in a vacuum oven at 80 °C for 6 h. Finally, the nanoporous carbon materials derived from zanthoxylum bungeanum peel (PAC-X) were obtained. The nanoporous carbon materials derived from zanthoxylum bungeanum seed (SAC-X) were also prepared with the same method.

The ash content of zanthoxylum bungeanum peel or seed was determined as follows: 2 g of zanthoxylum bungeanum peels or seeds were put into the muffle furnace and kept at 800 °C for 8 h with a heating rate of 5 °C min^−1^. After cooling to room temperature, the ash was weighed and recorded.

### 2.3. Structural and Physicochemical Characterizations of Nanoporous Carbon Materials

Microstructure: The microstructure was observed on a field-emission scanning electron microscope (FESEM, FEI QUANTA FEG 250) equipped with energy dispersive X-ray spectrometry along with a transmission electron microscopy (TEM, JEOL, 2100F, Tokyo, Japan).

Elemental compositions and valence state: The elemental compositions and valence state were detected and determined by X-ray photoelectron spectroscopy (XPS, VGESCALAB MKII).

Phase constitution and crystallinity: The phase constitution and crystallinity of the nanoporous carbon materials were determined using an X-ray diffractometer (Ultima IV, Rigaku, Japan) with Cu K_α_ radiation (λ = 0.15406 nm). The acceleration voltage of 40 kV and the emission current of 40 mA were adopted. The X-ray diffraction (XRD) patterns were collected within a diffraction angle (2θ) range from 10° to 90° at a step interval of 0.02° and a scanning rate of 10°/min.

Functional groups: The Fourier transform infrared (FT-IR) spectra were collected on an FT-IR spectrometer (Nicolet iS50, Thermo Fisher, Waltham, MA, USA) to characterize the functional groups of carbon materials. Prior to the functional group analysis, each sample was mixed with KBr according to a mass ratio of 1 to 200 and was ground adequately. Afterward, the sample was degassed and dried at 353 K and ca. 500 Pa for 6 h in a vacuum oven. Finally, the dried sample was pressed into a translucent wafer for testing.

Physisorption analysis: The specific surface area, absorbed volume, and pore-size distribution (PSD) of carbon materials were determined from N_2_ adsorption-desorption isotherms at 77.4 K using a surface characterization analyzer (SSA-6000, Beijing Builder, Beijing, China). The specific surface area (S_BET_) was calculated using the Brunauer-Emmett-Teller (BET) method in a range of relative pressure (P/P_0_) from 0.05 to 0.20 [27]. The Barrett-Joyner-Halenda (BJH) method was used to determine the pore size distribution (PSD) curves based on the adsorption isotherms but not the desorption isotherms [28]. This is because the interference of pseudopores appeared in the N_2_ desorption isotherms. The total pore volume (V_t_) was calculated at a relative pressure (P/P_0_) of 0.99 [29]. Prior to the physisorption analysis, all samples were degassed and dried at 573 K and ~500 Pa for 300 min to remove the moisture adsorbed on/in the porous structure.

Raman analysis: The graphitization degrees of carbon materials were determined from the Raman spectra recorded in the range from 500 to 2500 cm^−1^ with a Raman spectrometer (LabRAM HR Evolution, Horiba Jobin Yvon, Paris, France) using a He-Ne laser excitation wavelength of 632.8 nm [30].

### 2.4. Characterizations of Electrochemical Performances

Prior to the electrochemical tests, each working electrode was prepared through the following procedures: The nanoporous carbon material (4.0 mg), acetylene black (0.5 mg), and 5 wt.% PVDF ethanol solution (10.0 mg) were first mixed and then ground adequately to fabricate the electrode slurry. Subsequently, the electrode slurry was coated on one side of two pieces of continuous nickel foam with a size of 15 mm × 10 mm × 0.5 mm. After drying at 353 K for 30 min, nickel foams were pressed together under a proper pressure of ~0.5 MPa to ensure that the active material of ~2.0 mg was between two pieces of overlapping nickel foams. After that, all working electrodes were dried under a vacuum at 353 K for 12 h.

The electrochemical data, including cyclic voltammetry (CV) curves, galvanostatic charge-discharge (GCD) curves, and electrochemical impedance spectra (EIS), were collected on a CHI760E electrochemical workstation (Chenhua Instrument Shanghai Co., Ltd., Shanghai, China) using a three-electrode configuration, including a Hg/HgO electrode as the reference electrode and a platinum sheet as the counter electrode. CV and GCD curves were measured in a potential range from 0 to −1 V vs. Hg/HgO at various scan rates and current densities, respectively. EIS was collected in a frequency range from 10^5^ to 10^−2^ Hz with an AC amplitude of 5 mV. Additionally, the electrochemical capacitive performances of PAC-700 and SAC-600 were also evaluated in a symmetrical capacitor (SC). Moreover, two button-type supercapacitors were assembled for the stability tests. The stability of PAC-700 and SAC-600 were also evaluated by the constant-current (1 A g^−1^) charge-discharge tests, which were performed on a battery test system (LAND-CT2001A, Wuhan Jinnuo Electronics Co., Ltd., Wuhan, China).

In order to ensure the consistency of measurements and the comparability of data, the following items should be performed during all electrochemical tests: (a) a five-neck flask was used to ensure that all electrodes were in the same corresponding positions; (b) all electrolytes were 6.0 mol·L^−1^ KOH aqueous solution; (c) the working electrode and counter electrode kept a proper distance of ca. 2.5 cm to avoid an electrical short circuit; and (d) all electrodes and the five-neck flask should avoid any physical contact with each other.

The specific capacitance (C, F·g^−1^) for the working electrode, or SC, was calculated from the GCD curve according to Equation (1) [31].

(1) $$C=\frac{j\times t_{0}}{∆E}$$

Herein, *j* and *t*_0_ stand for the current density (A·g^−1^) and the discharge time (s), respectively. Δ*E* is the potential window (V), which is conditional on the thermodynamic stability of the electrodes and electrolyte.

The energy density (ED, Wh·kg^−1^) and the power density (PD, W·kg^−1^) of SC were successively calculated using Equations (2) and (3) [32,33].

(2) $$ED=\frac{1}{3.6}\times j\int_{0}^{t_{0}}Edt$$

(3) $$PD=3600\times \frac{ED}{t_{0}}$$

Herein, 1/3.6 and 3600 are the coefficients of dimensional conversion.

## 3. Results

### 3.1. Structural and Physicochemical Properties of Nanoporous Carbon Materials

**Figure 1:**
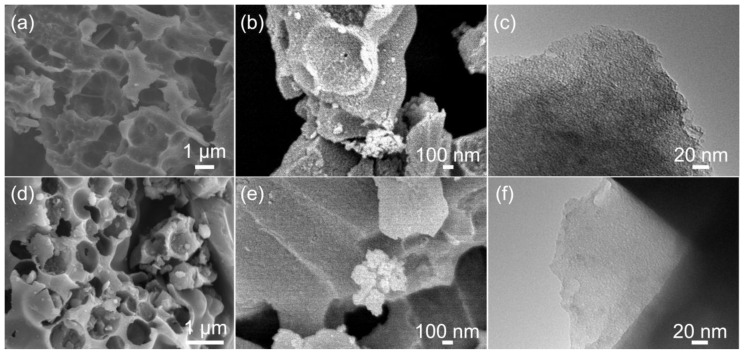
SEM images of (**a**,**b**) PAC-700 and (**d**,**e**) SAC-600; TEM images of (**c**) PAC-700 and (**f**) SAC-600.

Figure 1 shows the SEM and TEM images of PAC-700 and SAC-600. The uniform, open, and micrometer scale ligament-macropores structure forms in the PAC-700 and SAC-600 particles with the assistance of volatile organic components during the pyrolysis (Figure 1a,d). The thickness of the ligament in SAC-600 is larger than that in PAC-700, indicating the more abundant pore volume in PAC-700. Additionally, a tiny amount of mesopores also form in PAC-700 and SAC-600, resulting from the volatilization of organic components and the etching of KOH (Figure 1b,e). Moreover, plenty of micropores form in PAC-700 and SAC-600, resulting from the intercalation of K between the turbostratic graphite-like layers (Figure 1c,f) [34]. Hence, the hierarchical pore structure of PAC-700 and SAC-600 can provide abundant charge storage sites and convenient channels for ion migration.

**Figure 2:**
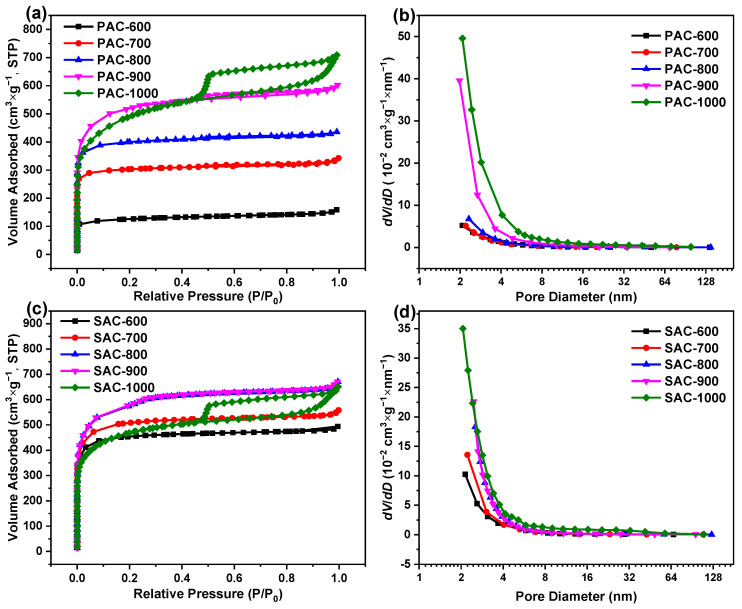
N_2_ adsorption−desorption isotherms of (**a**) PAC-X and (**c**) SAC-X, and PSD curves of (**b**) PAC-X and (**d**) SAC-X (X = 600, 700, 800, 900, and 1000).

In order to quantitatively evaluate the pore structure, N_2_ adsorption−desorption isotherms of PAC-X and SAC-X (X = 600, 700, 800, 900, and 1000) were measured, and the results are shown in Figure 2. Based on the latest classifications of physisorption isotherms and hysteresis loops by the International Union of Pure and Applied Chemistry (IUPAC) [35], all isotherms show the typical characteristics of type IV(a) isotherms with H4-type hysteresis loops for demonstrating the hierarchical porous structures with micropores, mesopores, and macropores for all samples (Figure 2a,c), which is in accord with SEM and TEM results (Figure 1). Concretely, a sharp adsorption (P/P_0_ < 0.01), an apparent adsorption (0.01< P/P_0_ < 0.1), a hysteresis loop (0.1 < P/P_0_ < 0.9), and an upward tendency (0.9 < P/P_0_) suggest substantial ultramicropores (<0.7 nm), considerable supermicropores (0.7 < d < 2.0 nm), and observable mesoporous and limited macropores, respectively.

Additionally, the volume of micropores obtains its maximal value at the activation temperature of 900 °C for all samples (Figure 2a,c), suggesting the micropores are prone to sintering above 900 °C. Compared to PAC-1000, the volume of micropores in SAC-1000 decreases more significantly than that of SAC-900, demonstrating that the micropores in SAC-1000 are more prone to sintering than those in PAC-1000. Furthermore, plenty of mesopores form at the activation temperature of 900 °C and 1000 °C for all samples (Figure 2a,c), implying the intensive etching of K_2_O and CO_2_ derived from K_2_CO_3_ at/above 900 °C. Further to this, the formation of mesopores in SAC-1000 is at the cost of sacrificing micropores, which result from sintering. As shown in Table 1, V_t_ of PAC-X increases with the increasing activation temperature and reaches the maximal value (1.10 cc g^−1^) at 1000 °C, while V_t_ of SAC-900 derives the maximal value (1.04 cc g^−1^), implying the increased pore volume of KOH activation is roughly equal to the decreased pore volume of sintering for SAC-1000 (1.01 cc g^−1^). Moreover, the upward tendency (0.9 < P/P_0_) is more significant with the increase in the activation temperature, demonstrating that the higher activation temperature contributes to the formation of macropores (significantly above 900 °C).

As depicted in Figure 2b,d, all PSD curves are without overlap and are arranged from bottom to top as PAC-X and SAC-X (X = 600, 700, 800, 900, and 1000), indicating the volume of mesopores increases with the increasing activation temperature for PAC-X and SAC-X (especially at/above 900 °C). It can be found from Table 1 that the changing trends of S_BET_ are similar to those of V_t_ for PAC-X and SAC-X_,_ implying all hierarchical porous structures are mainly composed of micropores, which can be proved by combining with Figure 2a,c. Additionally, the percentages of pore volume (V_micro_) and specific surface area (S_micro_) of micropores are higher than 63.0% and 74.0% for all samples, respectively, which also proves the above result. PAC-1000 and SAC-900 have the highest S_BET_, largest V_t_, and average pore diameters (D_a_) of ~1786.1 m^2^ g^−1^/~1.10 cc g^−1^/2.5 nm and ~1895.7 m^2^ g^−1^/~1.04 cc g^−1^/2.2 nm. PAC-700 and SAC-600 have the higher S_BET_, bigger V_t_, and larger D_a_ of ~970.6 m^2^ g^−1^/~0.53 cc g^−1^/2.2 nm and ~1557.9 m^2^ g^−1^/~0.71 cc g^−1^/2.0 nm, which are comparable to PAC-1000 and SAC-900. Especially SAC-600, it has such high S_BET_ (>1000 m^2^ g^−1^) and V_t_ (>0.50 cc g^−1^) even at the lowest temperature of 600 °C, which is very rarely found in other biomass-derived carbon. The unique hierarchical pore structure endows a good charge storage characteristic of PAC-700 (V_micro_, 86.5%; V_micro_, 9.2%; V_macro_, 4.3%) and SAC-600 (V_micro_, 89.1%; V_micro_, 8.0%; V_macro_, 2.9%).

**Figure 3:**
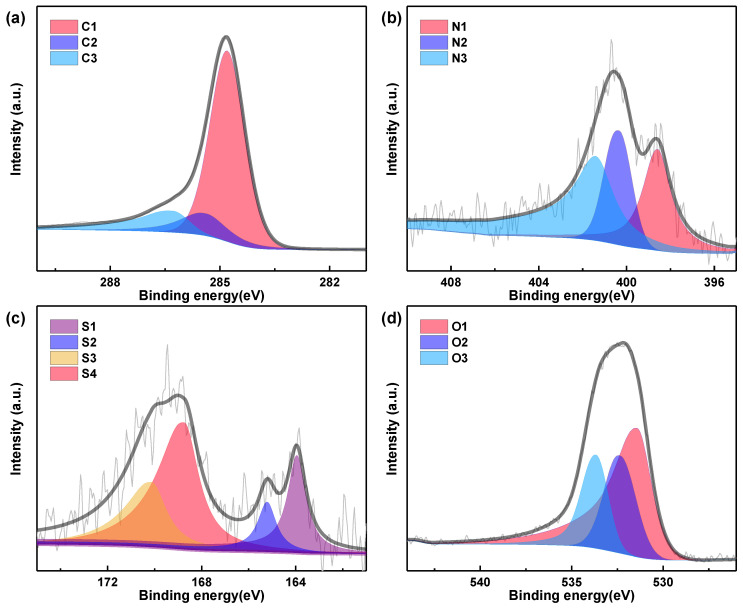
High-resolution XPS spectra of (**a**) C 1s, (**b**) N 1s, (**c**) S 2p and (**d**) O 1s of PAC-700.

**Figure 4:**
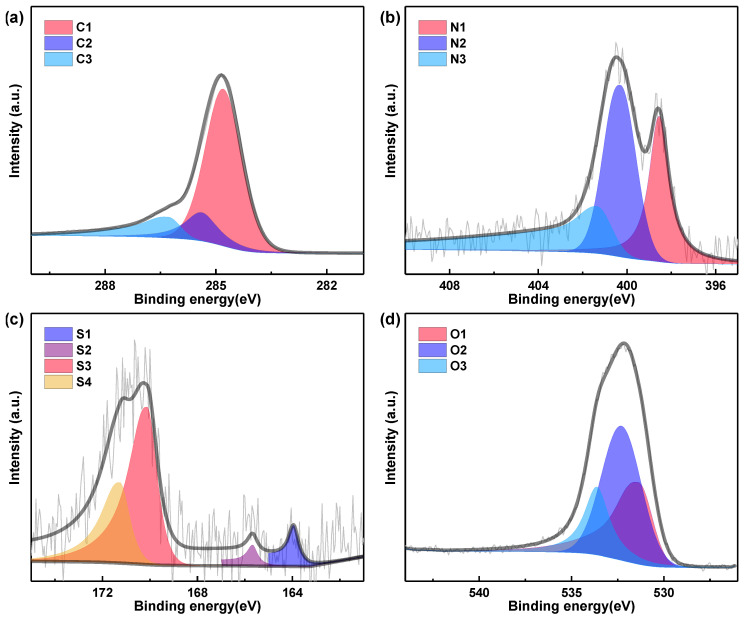
High-resolution XPS spectra of (**a**) C 1s, (**b**) N 1s, (**c**) S 2p, and (**d**) O 1s of SAC-600.

To determine the elemental species contents of PAC-700 and SAC-600, the XPS spectra were collected and fitted with thirteen Gaussian peaks (Figure 3, Figure 4 and Figure S1), and the analysis results are summarized in Table 2. The contents of C, N, S and O are ~80.84/~78.66 at%, ~3.50/~4.62 at%, ~0.61/~0.59 at%, and ~15.05/~16.13 at% for PAC-700/SAC-600.

All high-resolution C 1s spectra are typically divided into three components (Figure 3a and Figure 4a): C-I (C=C/C-C, ~284.8 eV), C-II (C–N/C–S/C-O, ~285.4 eV), and C-III (C=N/C=O, ~286.3 eV) [36]. All high-resolution N 1s spectra are decomposed into three components (Figure 3b and Figure 4b): N-6 (pyridinic N, ~398.6 eV), N-5 (pyrrolic N, ~400.3 eV), and N-Q (quaternary N, ~401.4 eV) [37,38]. As regards the high-resolution S 2p spectra (Figure 3c and Figure 4c), they are split into four configurations: S-I (C-S-C 2p_3/2_, ~164.0 eV,), S-II (C-S-C 2p_1/2_, ~165.2 eV), S-III (–C–SO_3_–C, ~168.8 eV), and S-IV (–C–SO_4_–C, ~170.1 eV) [39]. As for the high-resolution O 1s spectra (Figure 3d and Figure 4d), they are composed of three configurations: O-I (O=C, ~531.4 eV), O-II (O–C, ~532.4 eV), and O-III (HO-C, ~533.7 eV) [40].

In these groups, the heteroatoms (N, S, and O) containing functional groups are beneficial to improving the wettability/hydrophilicity of PAC-700 and SAC-600, resulting in an increase in electrochemical active sites and an enhancement of mass transfer. Concretely, SAC-600 (21.34 at%) is superior to PAC-700 (19.16 at%) in the content of heteroatoms (N, S, and O). N-6, N-5, and O-I groups can introduce extra pseudocapacitance through redox reactions. Specifically, the contents of N-6/N-5/O-I reach ~1.06/0.85/7.80 at% (PAC-700) and ~1.44/1.89/5.21 at% (SAC-600), and PAC-700 (9.71at%) is superior to SAC-600 (8.54 at%) in the content of N-5 and O-I groups. While one N-6/N-5 site can store three electrons and O-I can only store one electron [41]. Hence, the pseudocapacitance contribution of SAC-600 (15.20%) is higher than that of SAC-700 (13.53%). C-I and N-Q groups are conducive to the rapid transfer of electrons in PAC-700 and SAC-600, resulting in the enhancement of rate performances. Concretely, the contents of C-I/N-Q reach ~57.60/1.59 at% (PAC-700) and ~55.44/1.29 at% (SAC-600), and PAC-700 (59.19 at%) is superior to SAC-600 (56.73 at%) in the content of C-I and N-Q groups. Based on the above analysis, the unique functional groups endow a good charge storage characteristic of PAC-700 and SAC-600. Moreover, both zanthoxylum bungeanum peels and seeds contain extremely low amounts of ash due to almost 100% weight loss after calcination in the air.

**Figure 5:**
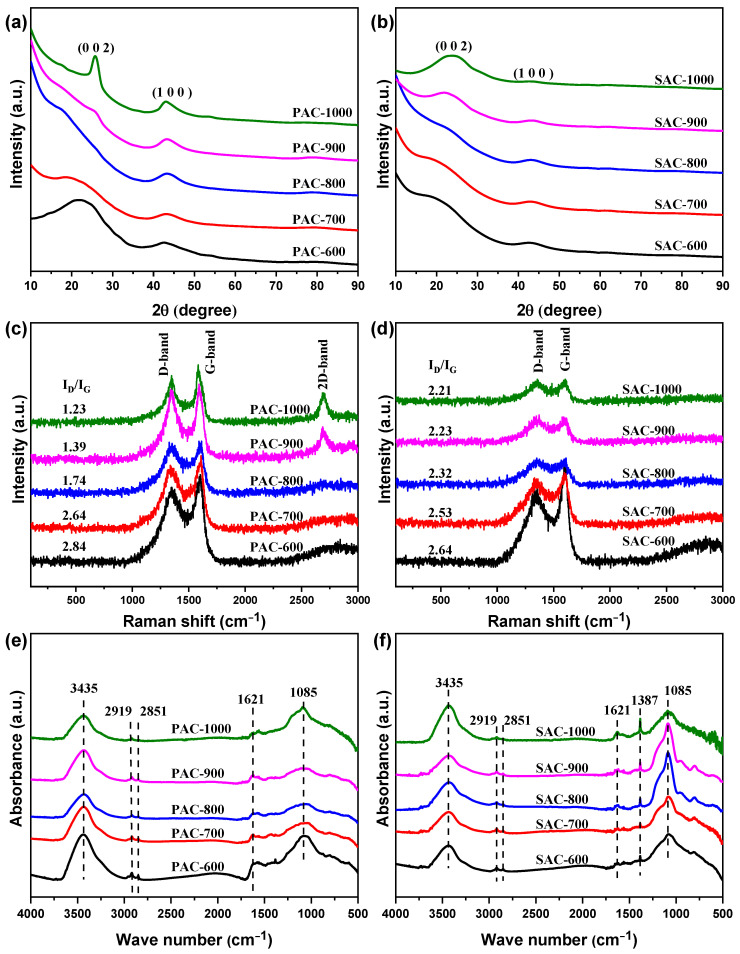
XRD patterns/Raman patterns/FT-IR spectra of (**a**/**c**/**e**) PAC-X and (**b**/**d**/**f**) SAC-X (X = 600, 700, 800, 900, and 1000).

All XRD patterns of PAC-X and SAC-X (X = 600, 700, 800, 900, and 1000) display two broad diffraction peaks at ~22.0–25.8° for the (002) crystal plane and ~42.9°–43.5° for the (100) crystal plane, and K-containing phases disappear (Figure 5a,b), indicating the turbostratic graphite-like structure for all samples. All full-width at the half of the maximum (FWHM) of PAC-X/SAC-X decreases from 10.6°/9.5° to 2.3°/7.2° for the (002) crystal plane; the size of the (002) crystal inner plane increases with the increasing activation temperature. This is similar to the (100) crystal plane. Significantly, the (002) crystal plane of PAC-1000 has the highest diffraction intensity and narrowest peak width, implying PAC-1000 derives the highest crystallinity. Additionally, the (002) crystal plane shifts from 42.9° to 43.1°, 43.3°, 43.5°, and 43.5° for PAC-X, and the (100) crystal plane shifts from 43.0° to 43.2°, 43.3°, 43.4°, and 43.5° for SAC-X, suggesting the interplanar spacing of the (002) and (100) crystal planes decreases with the increasing activation temperature. Furthermore, the diffraction intensity of the (100) crystal plane increases for PAC-X, while it decreases for SAC-X with the increasing activation temperature, implying the seeds of Zanthoxylum Bungeanum are more suitable for preparing carbon nanomaterials with low crystallinity. Based on the above analysis, the crystallinity increases and the defect content decreases with the increasing activation temperature. Hence, PAC-700 and SAC-600 have substantial defect sites that are conducive to charge storage. Moreover, PAC-700 and SAC-600 have larger interplanar spacings and smaller crystal inner plane sizes of (002) and (100), implying a higher S_BET_, which accords with the BET results. The substantial defect sites and higher S_BET_ endow the good charge storage characteristics of PAC-700 and SAC-600.

As depicted in Figure 5c,d, the I_D_/I_G_ values (namely the ratio of the D-band peak to the G-band peak in the integral intensity) decay continuously from ~2.84 to ~2.64, ~1.74, 1.39, and 1.23 for PAC-X (X = 600, 700, 800, 900, and 1000) [42]. The I_D_/I_G_ values drop constantly from ~2.64 to ~2.53, ~2.32, 2.23 and 2.21 for SAC-X (X = 600, 700, 800, 900, and 1000). These results are indicative of the increase in the graphitization degree of PAC-X and SAC-X and the decrease in the content of defect sites with the increasing activation temperature, which accords with the XRD results. Additionally, prominent D-band peaks appear in the Raman spectra of PAC-900 and PAC-1000. The above results indicate that the zanthoxylum bungeanum peel is more likely to be graphitized than the zanthoxylum bungeanum seed, which is also in line with the XRD results. Based on the above analysis, the substantial defect sites are conducive to providing abundant charge storage sites and improving capacitance.

As shown in Figure 5e,f, the peaks in the FT-IR spectra are assigned to the following functional groups: the O-H or N-H stretching vibration (~3435 cm^−1^), C-H stretching vibration (~2919 and ~2851 cm^−1^), C=O stretching vibration (1621 cm^−1^), O-H in-plane bending vibration (1387 cm^−1^) and C-O or C-S-C stretching vibration (1085 cm^−1^) [43]. The functional group components agree with the XPS results. SAC-X is more significant than PAC-X in the intensity of the peak at 1085 cm^−1^, which is also in line with XPS results (S-I + S-II + O-II: 4.07 at%/PAC-700 and 7.77 at%/SAC-600). Therefore, all prepared carbon materials contain heteroatoms O and N, which may contribute to the generation of pseudocapacitance. Thus, the unique functional groups endow a good charge storage characteristic of PAC-700 and SAC-600.

### 3.2. Electrochemical Performances of Nanoporous Carbon Materials

**Figure 6:**
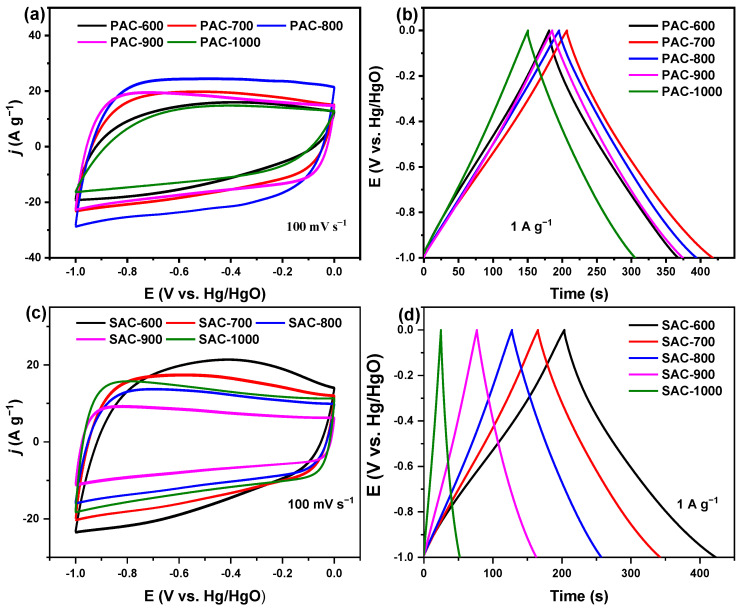
CV curves recorded at a scan rate of 100 mV s^−1^ for (**a**) PAC-X and (**c**) SAC-X, and GCD curves measured at a current density of 1 A g^−1^ for (**b**) PAC-X and (**d**) SAC-X (X = 600, 700, 800, 900, and 1000).

All CV curves of PAC-X and SAC-X (X = 600, 700, 800, 900, and 1000) present the pseudo-rectangle without the prominent redox peaks (except PAC-900 and PAC-1000) and the sizeable electrochemical response currents (Figure 6a,c), indicating the dominant electric double-layer capacitance (EDLC) behavior. The distortion of each CV curve is mainly due to the longer response time for charge and discharge, along with the pseudocapacitance.

All GCD curves of PAC-X and SAC-X (X = 600, 700, 800, 900, and 1000) are basically symmetrical pseudo-triangles (Figure 6b,d), also suggesting the dominance of EDLC behavior and good electrochemical reversibility. The slight distortion of GCD curves results from the pseudocapacitance produced by the N-6, N-5, and O-I groups. PAC-700 and SAC-600 have the most extended discharge times of ~211.0 and ~219.7 s in one GCD cycle, demonstrating the highest specific capacitance of ~211.0 (PAC-700) and ~219.7 (SAC-600) F g^−1^ at 1 A g^−1^, which is higher than other reported data (Table S1). The specific capacitances normalized by SBET are ~21.7 (PAC-700) and ~14.1 (SAC-600) μF cm^−2^ for PAC-700 and SAC-600, which are 2.2 and 1.4 times that of the commercial activated carbon (10 μF cm^−2^) [44].

**Figure 7:**
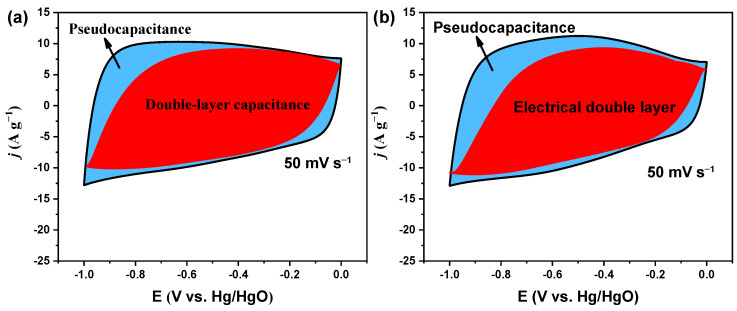
EDLC and pseudocapacitance contents of (**a**) PAC-700 and (**b**) SAC-600.

In order to determine the contribution ratios of EDLC and pseudocapacitance, the EDLC and pseudocapacitance are derived by the Dunn method [45]. To detail, the relationship between the current (*i*) and scan rate (*v*) is subject to *i*(*v*) = *k*_1_*v* + *k*_2_*v*^1/2^ during the electrochemical reaction process. Thus, the combination of EDLC contribution (*k*_1_*v*) and pseudocapacitance (*k*_2_*v*^1/2^) can be divided via a linear fitting method. The contribution ratio of pseudocapacitance for SAC-600 (26%) is higher than that of PAC-700 (20%) (Figure 7a,b), which is in line with XPS results. The above results indicate that more defect sites in SAC-600 can introduce more pseudocapacitance.

**Figure 8:**
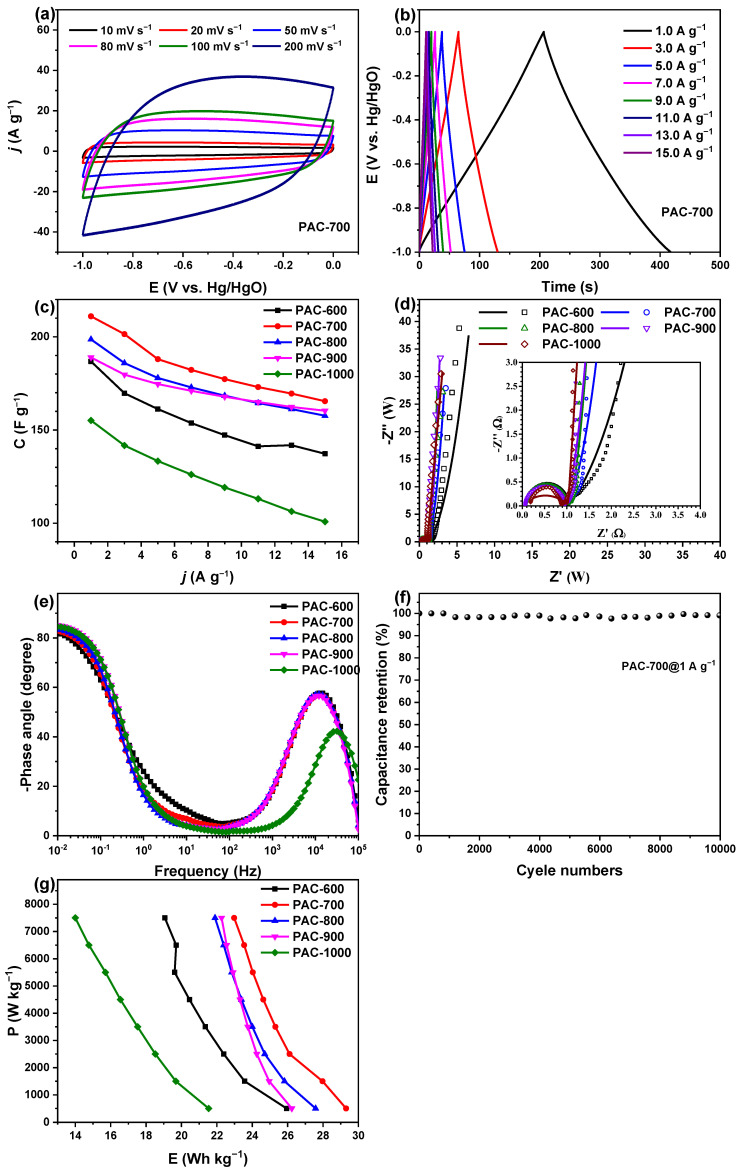
(**a**) CV curves of PAC-700 at 10, 20, 50, 80, 100, and 200 mV s^−1^. (**b**) GCD curves of PAC-700. (**c**) Specific capacitances of PAC-X at 1.0, 3.0, 7.0, 9.0, 11.0, 13.0, and 15 A g^−1^. (**d**) Nyquist plots of PAC-X with an enlarged view of the high-frequency region as the inset. (**e**) Bode plots of PAC-X. (**f**) Cyclic stability of the PAC-700 at a current density of 1 Ag^−1^. (**g**) Ragone plots of PAC-X (X = 600, 700, 800, 900, and 1000).

**Figure 9:**
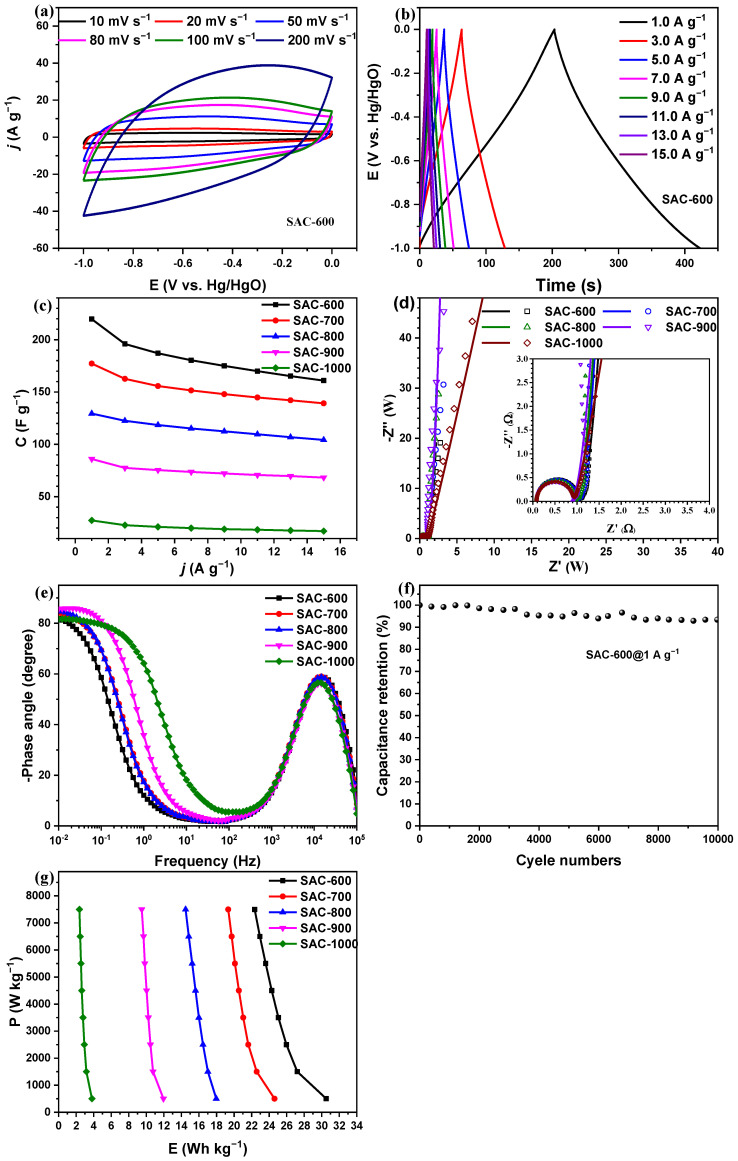
(**a**) CV curves of SAC-600 at 10, 20, 50, 80, 100, and 200 mV s^−1^. (**b**) GCD curves of SAC-600. (**c**) Specific capacitances of SAC-X at 1.0, 3.0, 7.0, 9.0, 11.0, 13.0, and 15 A g^−1^. (**d**) Nyquist plots of SAC-X with an enlarged view of the high-frequency region as the inset. (**e**) Bode plots of SAC-X. (**f**) cyclic stability of the SAC-700 at a current density of 1 Ag^−1^. (**g**) Ragone plots of SAC-X (X = 600, 700, 800, 900, and 1000).

Figure 8a and Figure 9a show the CV curves of PAC-700 and SAC-600 at different scanning rates of 10, 20, 50, 80, 100, and 200 mV s^−1^, respectively. All CV curves of PAC-700 and SAC-600 display pseudo-rectangle shapes, and the response currents rise significantly with the increasing scanning rates, suggesting the dominance of EDLC behavior. The response currents of PAC-700 are similar to those of SAC-600. Figure 8b and Figure 9b display the GCD curves of PAC-700 and SAC-600 at different current densities of 1.0, 3.0, 5.0, 7.0, 9.0, 11.0, 13.0, and 15 A g^−1^, respectively. All GCD curves of PAC-700 and SAC-600 show the symmetrical pseudo-triangles without apparent IR drop, further suggesting the dominance of EDLC behavior, good electrochemical reversibility, and lower internal series resistance, which endows the good rate performances.

Figure 8c and Figure 9c show the rate performances of PAC-X and SAC-X (X = 600, 700, 800, 900, and 1000). The specific capacitances of PAC-700 and SAC-600 are respectively higher than those of other PAC-X and SAC-X at all current densities, suggesting PAC-700 and SAC-600 have separately the highest specific capacitances among PAC-X and SAC-X and the optimal activation temperatures are respectively 700 °C and 600 °C for zanthoxylum bungeanum peels and seeds. Concretely, the specific capacitances of PAC-700 are separately 211.0, 201.4, 188.0, 182.2, 177.3, 173.0, 169.5, and 165.5 F g^−1^ at 1.0, 3.0, 5.0, 7.0, 9.0, 11.0, 13.0, and 15 A g^−1^ with a retention rate of 78.4%. The specific capacitances of SAC-600 are separately 219.7, 196.0, 187.0, 180.4, 175.0, 170.0, 165.2, and 161.0 F g^−1^ at 1.0, 3.0, 5.0, 7.0, 9.0, 11.0, 13.0, and 15 A g^−1^ with a retention rate of 73.3%, which is comparable to that of PAC-700 (78.4%). Specifically, the curves of rate performance for SAC-X (X = 600, 700, 800, 900, and 1000) are without overlap (Figure 9c). SAC-600 derives its abundant pore structures and high specific capacitance (>200 F g^−1^) at such a low activation temperature, which is rare. Thus, the internal factors are worthy of in-depth research.

The Nyquist plots of PAC-X and SAC-X (X = 600, 700, 800, 900, and 1000) are shown in Figure 8d and Figure 9d. A semicircle appears in the medium frequency region of each Nyquist plot, and the diameter of the semicircle stands for the charge transfer resistance between the electrolyte and electrode (R_ct_). Each Nyquist plot displays a straight line in the low-frequency region, and the greater slope of the straight line represents the faster ion diffusion on the electrode material. The intercept on the real axis in the high-frequency region corresponds to the ohmic resistance from the electrolyte and the contact resistance at the interface between the active material and the collector (R_s_). Obviously, all values of R_s_ for PAC-X and SAC-X are close to 0.1 Ω, which shows that the device has a lower series resistance, a good conductivity of the electrolyte, and a lower internal resistance of the electrode. Additionally, all values of R_ct_ for PAC-X and SAC-X are less than 1 Ω, indicating good charge transfer characteristics. Furthermore, the vertical straight line in the low-frequency region indicates the low resistance of ion diffusion inside the electrode material. Concretely, the slopes of straight lines for PAC-X increase, while the slopes of straight lines for SAC-X decrease with the increasing activation temperature. Based on the above analysis, the low R_ct_, low R_s_, and high slope of the straight line endow the characteristics of fast charge storage in PAC-700 and SAC-600.

Figure 8e and Figure 9e show the Bode plots of PAC-X and PBC-X (X = 600, 700, 800, 900, and 1000), namely the relationship between phase angle and frequency. When the phase angle equals −90°, the capacitor is a pure capacitor circuit. In real capacitors, the phase angle deviates from −90° due to the complex interaction between the electrolyte and the electrode materials. Hence, the closer the phase angle is to −90°, the more similar the device is to a pure capacitor. Concretely, the phase angles at 10^−2^ Hz of PAC-X (X = 600, 700, 800, 900, and 1000) are ~82.2, ~82.8, ~83.5, ~85.2, and ~84.6°, respectively. The phase angles of SAC-X (X = 600, 700, 800, 900, and 1000) are ~81.8, ~84.1, ~85.0, ~85.1, and ~81.6°, respectively. The phase angles of all samples are above 80.0°, indicating that electrolyte ions diffuse rapidly in the framework of nanoporous carbon materials. Additionally, the peak frequency stands for the charge storage frequency. The peak frequencies of all samples are near 2 × 10^4^ Hz, except for PAC-1000, whose peak frequency is 3 × 10^4^ Hz. The result accords with the largest slope of the straight line (Figure 8d and Figure 9d).

Figure 8f and Figure 9f depict the capacitance retention rates of PAC-700//PAC-700 and SAC-600//SAC-600 symmetrical supercapacitors at a current density of 1 A g^−1^. PAC-700//PAC-700 and SAC-600//SAC-600 symmetrical supercapacitors have good cycle stability with capacitance retention rates of ~99.1% (former, 47.2 F g^−1^) and ~93.4% (latter, 44.6 F g^−1^) after 10,000 GCD cycles. Figure 8g and Figure 9g show the Ragone diagram of PAC-X and SAC-X in 6.0 M KOH. PAC-700 has a 29.3 Wh kg^−1^ energy density at a power density of 500 W kg^−1^, and the energy density can still reach 22.9 Wh kg^−1^ at a higher power density of 7500 W kg^−1^. SAC-600 has 30.5 Wh kg^−1^ at 500 W kg^−1^, and the energy density can still reach 22.4 Wh kg^−1^ at 7500 W kg^−1^. The PAC-700 and SAC-600 have high comparability with the previously reported biomass-based carbon materials [46,47,48]. Therefore, PAC-700 and SAC-600 can be used as the electrode materials for supercapacitors. Considering the cost, SAC-600 has greater application potential. Their excellent electrochemical properties, especially the combination of high power-energy density, can meet the needs of high-performance supercapacitors.

## 4. Conclusions

In summary, we successfully prepared the carbon materials with high specific capacitance from zanthoxylum bungeanum peels and seeds via pyrolysis and equivalent KOH activation at low temperatures of 700 °C and 600 °C. The abundant micropores, moderate mesopores, limited macropores, substantial heteroatoms (N, S, and O) containing functional groups, and plentiful defect sites are highly favored for the enhanced electrochemical active sites/interfaces and rapid ion transport towards efficient charge storage. The PAC-700 and SAC-600 exhibit large capacities of ~211.0 and ~219.7 F g^−1^ at 1.0 A g^−1^ within 6 M KOH at a three-electrode system. Additionally, the assembled PAC-700//PAC-700 and SAC-600//SAC-600 symmetrical supercapacitors obtain a high energy density of 22.9 and 22.4 Wh kg^−1^ at 7500 W kg^−1^ and electrochemical stability over 10,000 cycles at 1.0 A g^−1^. These features highly embody the potential of biomass-derived nanoporous carbon materials towards supercapacitors and beyond.

## Figures and Tables

**Table 1:** Porosity parameters of PAC-X and SAC-X (X = 600, 700, 800, 900, and 1000).

Sample	S_BET_ [m^2^ g^−1^]	S_micro_ [m^2^ g^−1^]	V_t_ [cc g^−1^]	V_micro_ [cc g^−1^]	V_meso_ [cc g^−1^]	D_a_ [nm]
PAC-600	436.5	389.3	0.25	0.20	0.03	2.3
PAC-700	970.6	877.4	0.53	0.46	0.05	2.2
PAC-800	1325.8	1215.1	0.68	0.61	0.05	2.0
PAC-900	1926.3	1658.2	0.94	0.76	0.15	1.9
PAC-1000	1786.1	1366.5	1.10	0.69	0.39	2.5
SAC-600	1557.9	1432.8	0.71	0.63	0.06	2.0
SAC-700	1806.7	1621.8	0.87	0.76	0.08	1.9
SAC-800	1857.4	1587.1	1.04	0.84	0.15	2.3
SAC-900	1895.7	1597.5	1.04	0.83	0.16	2.2
SAC-1000	1612.5	1198.7	1.01	0.67	0.28	2.5

**Table 2:** The elemental species contents of PAC-700 and SAC-600 derived from fitted XPS data.

Binding Energy (eV)	PAC-700	Ration (at%)	SAC-600	Ratio (at%)
	C 1s	80.84	C 1s	78.66
284.80	C-I	57.60	C-I	55.44
285.39	C-II	7.99	C-II	11.87
286.30	C-III	15.34	C-III	11.35
	N 1s	3.50	N 1s	4.62
398.58	N-6	1.06	N-6	1.44
400.34	N-5	0.85	N-5	1.89
401.41	N-Q	1.59	N-Q	1.29
	S 2p	0.61	S 2p	0.59
163.95	S-I	0.21	S-I	0.23
165.22	S-II	0	S-II	0
168.74	S-III	0.40	S-III	0.36
170.12	S-IV	0	S-IV	0
	O 1s	15.05	O 1s	16.13
531.42	O-I	7.80	O-I	5.21
532.35	O-II	3.86	O-II	7.54
533.68	O-III	3.39	O-III	3.38

## Data Availability

The data presented in this work are available on request from the corresponding authors.

## Supplementary Materials

The following supporting information can be downloaded at: https://www.mdpi.com/article/10.3390/nano14100836/s1, Figure S1: XPS survey spectra of PAC-700 and SAC-600; Table S1: Comparisons of SBET and specific capacitance with the reported data. (References [49,50,51,52,53,54] are cited in Supplementary Materials).
