# Supplementary material for: Nanoporous Carbon Materials Derived from Zanthoxylum Bungeanum Peel and Seed for Electrochemical Supercapacitors
Source: Nanomaterials (Basel). 2024 May 9;14(10):836. doi: 10.3390/nano14100836 (PMC11124505; doi:10.3390/nano14100836)
Supplement: Supplementary file 1 [file nanomaterials-14-00836-s001.zip › nanomaterials-2978200-supplementary.pdf]

Supporting Information for:

Nanoporous Carbon Materials Derived from Zanthoxylum Bungeanum Peel and Seed for Electrochemical Supercapacitors

Peng Jia[1], Ziming Wang[1], Xinru Wang[1], Ke Qin[1], Jiajing Gao[1], Jiazhen Sun[1], Guangmei Xia[1], Tao Dong[1], Yanyan Gong[1], Zhenjiang Yu[1], Jinyang Zhang[1,2,*], Honglei Chen[1,**], Shengdan Wang[1,†]

[1]Key Laboratory of Pulp and Paper Science & Technology of Ministry of Education/Shandong Province, State Key Laboratory of Biobased Material and Green Papermaking, Faculty of Light Industry, Qilu University of Technology (Shandong Academy of Sciences), Jinan 250353, P. R. China; skl_jiapeng@qlu.edu.cn (P.J.); 202291040062@stu.qlu.edu.cn (Z.W.); 202291040040@stu.qlu.edu.cn (X.W.); 202182060027@stu.qlu.edu.cn (K.Q.); 202182040004@stu.qlu.edu.cn (J.G.); jiazhensun@qlu.edu.cn (J.S.); gmxia@qlu.edu.cn (G.X.); dongt_skl@qlu.edu.cn (T.D.); 531995@qlu.edu.cn (Y.G.); yuzhen-jiang@hit.edu.cn (Z.Y.); zhangjy@nankai.edu.cn (J.Z.); wsd6849@qlu.edu.cn (S.W.); 501220@qlu.edu.cn (H.C.)

[2]Key Laboratory of Advanced Energy Materials Chemistry (Ministry of Education), Renewable Energy Conversion and Storage Center (RECAST), College of Chemistry, Nankai University, Tianjin 300071, P. R. China; zhangjy@nankai.edu.cn (J.Z.)

*The First Corresponding Author: zhangjy@nankai.edu.cn (J.Z.)

**The Second Corresponding Author: 501220@qlu.edu.cn (H.C.)

†The author contributed equally to this work with the corresponding authors: wsd6849@qlu.edu.cn (S.W.)

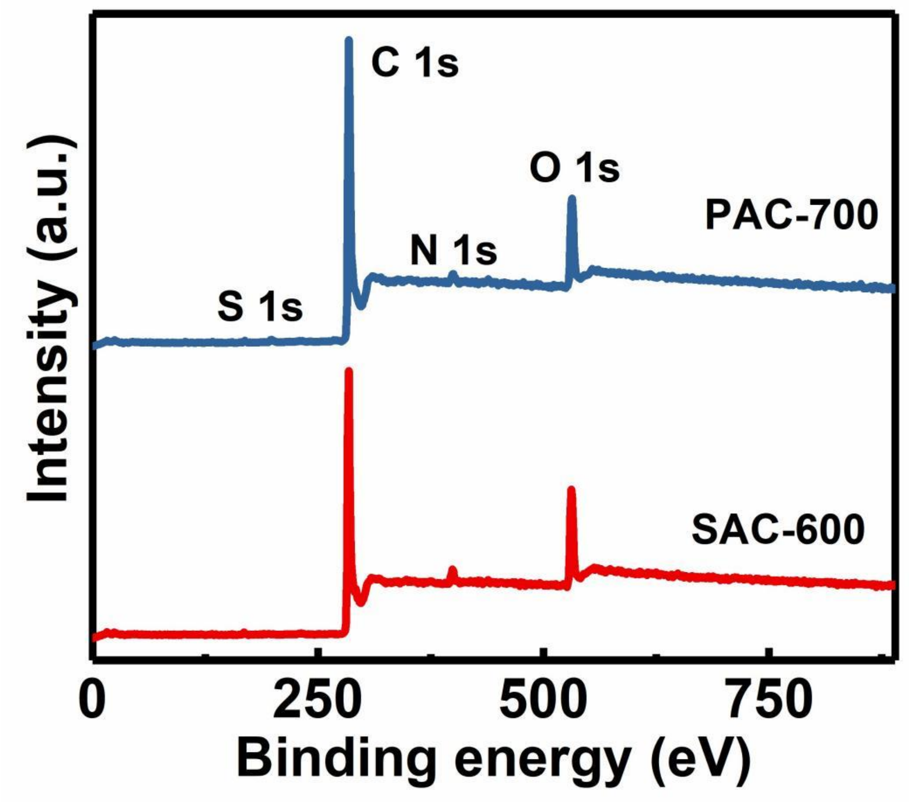

Figure S1 XPS survey spectra of PAC-700 and SAC-600.

Table S1 Comparisons of S_{BET} and specific capacitance with the reported data.

Biomass	S_{BET} ($m^2 \cdot g^{-1}$)	Specific capacitance ($F \cdot g^{-1}$)	Electrolyte	Current density ($A \cdot g^{-1}$)	References
PAC-700	970.6	211.0	6 M KOH	1.0	This work
SAC-600	1557.9	219.7	6 M KOH	1.0	This work
Pine sawdust	2331	175.6	6 M KOH	0.5	[S1]
Casein	2212	177	6 M KOH	0.5	[S2]
Rice husk	1183	148	6 M KOH	0.2	[S3]
Licorice Residues	2186	221	6 M KOH	0.5	[S4]
Camphor tree grains	470	384	6 M NaOH	1.0	[S5]
Rice straw	3345	365	2 M NaCl	0.5	[S6]

References

S1. Quan, C.; Su, R.; Gao, N. Preparation of activated biomass carbon from pine sawdust for supercapacitor and CO_2 capture. *Int. J. Energy Res.* **2020**, *44*, 4335−4351.
DOI: https://doi.org/10.1002/er.5206

S2. Singh, G.; Bahadur, R.; Ruban, A.M.; Davidraj, J.M.; Su, D.; Vinu, A. Synthesis of functionalized nanoporous biocarbons with high surface area for CO_2 capture and supercapacitor applications. Green Chem. **2021**, *23*, 5571−5583.
DOI: https://doi.org/10.1039/D1GC01376A

S3. Liu, Y.; Tan, H.; Tan, Z.; Cheng, X. Rice husk derived capacitive carbon prepared by one-step molten salt carbonization for supercapacitors. *J. Energy Storage* **2022**, *55*, 105437.
DOI: https://doi.org/10.1016/j.est.2022.105437

S4. Zhou, Q.; Li, H.; Jia, B.; Dang, Y.; Zhang, G. One-pot synthesis of porous carbon from Chinese medicine residues driven by potassium citrate and application in supercapacitors. *J. Anal. Appl. Pyrolysis* **2023**, *170*, 105894.
DOI: https://doi.org/10.1016/j.jaap.2023.105894

S5. Hao, J.; Wang, B.; Xu, H.; Du, J.; Wu, C.; Qin, W.; Wu, X. Interfacial regulation of biomass-derived carbon towards high-performance supercapacitor. *J. Energy Storage* **2024**, *86*, 111301.
DOI: https://doi.org/10.1016/j.est.2024.111301

S6. Dutta, R.K.A. A comparative study on porous activated carbon derived from waste biomass with varying oxygen functionalities as supercapacitor electrodes. *Energy Storage*, **2024**, *6*, e554.
DOI: https://doi.org/10.1002/est2.554
